# HDL as Bidirectional Lipid Vectors: Time for New Paradigms

**DOI:** 10.3390/biomedicines10051180

**Published:** 2022-05-20

**Authors:** María Luna-Luna, Eric Niesor, Óscar Pérez-Méndez

**Affiliations:** 1 Department of Molecular Biology, Instituto Nacional de Cardiología “Ignacio Chávez”, Mexico City 14080, Mexico; maria.luna@cardiologia.org.mx; 2 Hartis Pharma, 13C Chemin de Bonmont, 1260 Nyon, Switzerland; eniesor@hartispharma.com; 3 School of Engineering and Sciences, Tecnológico de Monterrey, Campus Ciudad de México, Mexico City 14380, Mexico

**Keywords:** apolipoprotein AI, diabetes mellitus, sphingomyelin, cholesterol, wound healing, endothelial cell, insulin secretion, embryogenesis, cancer

## Abstract

The anti-atherogenic properties of high-density lipoproteins (HDL) have been explained mainly by reverse cholesterol transport (RCT) from peripheral tissues to the liver. The RCT seems to agree with most of the negative epidemiological correlations between HDL cholesterol levels and coronary artery disease. However, therapies designed to increase HDL cholesterol failed to reduce cardiovascular risk, despite their capacity to improve cholesterol efflux, the first stage of RCT. Therefore, the cardioprotective role of HDL may not be explained by RCT, and it is time for new paradigms about the physiological function of these lipoproteins. It should be considered that the main HDL apolipoprotein, apo AI, has been highly conserved throughout evolution. Consequently, these lipoproteins play an essential physiological role beyond their capacity to protect against atherosclerosis. We propose HDL as bidirectional lipid vectors carrying lipids from and to tissues according to their local context. Lipid influx mediated by HDL appears to be particularly important for tissue repair right on site where the damage occurs, including arteries during the first stages of atherosclerosis. In contrast, the HDL-lipid efflux is relevant for secretory cells where the fusion of intracellular vesicles drastically enlarges the cytoplasmic membrane with the potential consequence of impairment of cell function. In such circumstances, HDL could deliver some functional lipids and pick up not only cholesterol but an integral part of the membrane in excess, restoring the viability of the secretory cells. This hypothesis is congruent with the beneficial effects of HDL against atherosclerosis as well as with their capacity to induce insulin secretion and merits experimental exploration.

## 1. Introduction

High-density lipoproteins (HDL) are complex macromolecules consisting of amphipathic lipids on the surface (free cholesterol and phospholipids) and non-polar lipids in the core (cholesteryl esters and triglycerides) [1,2]. The complex is stabilized by proteins named apolipoproteins, such as apolipoprotein (apo) AI, apo AII, apo AIV, the apo Cs, apo D, apo E, apo M and apo J [1]; apo AI and apo AII are the most abundant proteins, with the former representing up to 70% of the HDL protein mass [1,2].

The inverse relationship between the concentration of HDL cholesterol (HDL-C) and the risk of coronary artery disease is well known [1,3] and presupposes a causal relationship. For many years, HDL have been considered anti-atherogenic particles due to their ability to promote cholesterol efflux and their anti-oxidant, anti-aggregating, anti-coagulant, and anti-inflammatory properties [1,2]. The main anti-atherosclerotic mechanism associated with HDL is the reverse cholesterol transport (RCT), in which cholesterol from peripheral tissues is picked up by HDL and ultimately returned to the liver for its excretion or recycling [1,2,3].

RCT seems in agreement with most of the epidemiological observations regarding the relationship between cardiovascular risk and plasma levels of HDL-C. Early interpretations considered HDL-C plasma concentrations as a marker of the number of HDL particles and the amount of cholesterol efflux from tissues [3]. Today, the concept of HDL functionality, particularly its capacity to promote cholesterol efflux (the first step of RCT), has replaced HDL-C as a biomarker of coronary artery disease (CAD) risk [4]. In addition, there are some reports of CAD patients with high levels of HDL-C but with poor levels of phospholipids, which results in a decreased cholesterol efflux capacity (CEC) [5], suggesting that the quality of HDL is also a determinant of HDL function. Although CEC may predict CAD [6], this in vitro test does not seem to be independent of HDL-C levels in untreated CAD patients and in well-matched controls [6] or of inflammation markers such as high-sensitivity C-reactive protein [7]. It is also unknown whether CEC is, in fact, a good biomarker of the whole RCT.

Importantly, therapies designed to raise HDL-C, despite increasing HDL-induced CEC [6,8], failed to reduce cardiovascular risk. In addition, studies of mutations in genes related to the metabolism or structure of HDL revealed that very low HDL levels do not necessarily lead to increased cardiovascular risk [1,3]. These observations indicate that RCT may not explain the cardioprotective role of HDL. Other properties of HDL have been suggested as responsible for their anti-atherogenic potential [1]. It is obvious that the role of these lipoproteins is essential for life because HDL has been conserved throughout evolution, from cartilaginous fish to humans [9]. For the same reason, it is also apparent that their original biological role may not be to protect arteries against atherosclerosis via the RCT. Therefore, based on the actual evidence, it is time to reconsider the physiological function of HDL and establish new paradigms that must also explain the overall beneficial properties of these lipoproteins. This manuscript reviews the existing information supporting the idea that HDL is a lipid vector required to deliver lipids from the liver to tissues and vice versa. Such activity is particularly important for tissue repair right at the moment and at the site where the damage occurs. We include some evolutive evidence about the origin of lipoproteins and discuss some circumstances where tissue damage and a drop in HDL levels seem to be associated with noxious outcomes. We further review the capacity of HDL to promote insulin secretion, which could be regulated by lipid efflux that becomes abnormal in the β-cell membrane after the fusion of insulin secretory vesicles.

## 2. The Failure of Reverse Cholesterol Transport as a Predictive Paradigm

**Figure 1:**
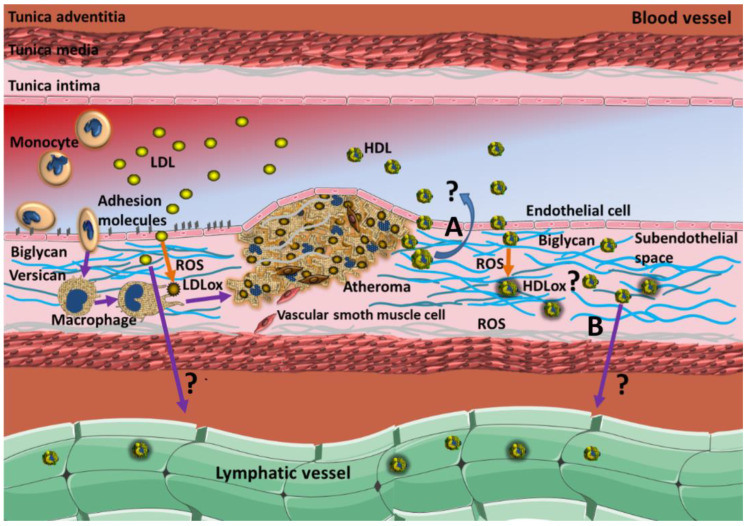
Proposed role of LDL and HDL on atheroma formation and progression. In the onset of endothelial dysfunction, LDLs reach the subendothelial space, where they become oxidized, induce chemiotaxis for monocytes, and are phagocytized by macrophages inducing the inflammatory process and lipid streak formation. By contrast, HDL has been proposed to cross the endothelial barrier and recover the cholesterol from foam cells (cholesterol efflux, not shown in the image). To continue with the next steps of the reverse cholesterol transport, HDL must leave the subendothelial space; for this, the direct return of HDL to blood circulation (**A**) is not physicochemically favored against hydraulic pressure and concentration gradient. The lymphatic circulation is the most plausible alternative for HDL to abandon the blood vessel (**B**), but this way out is also accessible for LDL. LDL seems to be retained in the tissue by interacting with versican and biglycan, which also interacts and probably retains HDL in the subendothelial space, impeding the next steps of RCT.

Many epidemiological studies have demonstrated the inverse relationship between HDL-C levels and the development of coronary artery disease (CAD) [10,11,12]. The main anti-atherogenic mechanism of HDL has been attributed to RCT. This intravascular pathway has been proposed to begin in the liver and the intestines by synthesizing discoid particles containing lipid-poor apo AI [13]. These premature HDLs acquire phospholipids and cholesterol from tissues via the ATP-binding cassette, subfamily A, member 1 (ABC-A1) transporter to become spherical HDL [14,15]. Cholesterol uptake by these particles could be relevant when they cross the endothelial barrier and promote cholesterol efflux from cholesterol-loaded foam cells, reducing their lipid content and delaying atheroma formation (Figure 1). At this point, it is easy to postulate that HDL crosses the endothelial barrier by transcytosis [16], as also described for low-density lipoproteins (LDL) [17]. However, once HDL has promoted cholesterol efflux from foam cells, the lipoproteins’ mechanisms leave the subendothelial space to continue the next RCT steps, i.e., cholesterol esterification, triglyceride exchange, and internalization by the liver, which are worth further consideration. First, the lower hydraulic pressure in the interstitial space compared with the coronary blood pressure and the potential adhesion of HDL to proteoglycans [18,19] are incompatible with a spontaneous exit of HDL from the subendothelial space back to the blood. Moreover, to our knowledge, there is no described active transport of HDL in endothelial cells, i.e., against the HDL concentration gradient. Therefore, the way out for HDL from the subendothelial space is the lymphatic circulation (Figure 1) [16,20]; unfortunately, lymphangiogenesis and cardiac lymphatic circulation have not been considered part of RCT. The more efficient the lymphatic circulation, the better cholesterol efflux would be. In this context, whatever the way out of HDL from the subendothelial space is, LDL would also have the possibilities to exit from this space by the same means promoting cholesterol efflux, thus stressing the validity of RCT driven only by HDL.

Independently of the path used by HDL to leave the subendothelial space, it is accepted that HDL drives cholesterol from the tissues back to the hepatocytes mainly by SR-BI internalization [21] but also via the F(1)-ATPase/P2Y(13) complex [22,23]. Then, a fraction of the total cholesterol pool reaches the small intestine [24]. Most intestinal cholesterol is of bile origin, whereas a minor fraction is hepatic-independent, known as transintestinal cholesterol excretion (TACE). TACE seems to also be independent of HDL [25], but this issue remains controversial [26]. Instead, apo B-containing lipoproteins may drive the cholesterol to the basolateral membrane of enterocytes [24]. Cholesterol is further secreted by the apical membrane via ABCG5/ABCG8 heterodimers to the intestinal lumen [27,28,29]. Then, about 50% of the intestinal cholesterol is excreted in feces [30], whereas the remaining fraction is selectively taken up by the Niemann–Pick C1 Like 1 protein [31]. Most intestinal cholesterol is transported to the blood via the lymphatic system, packed in chylomicrons [30]. Intestinal HDL seems to be implicated in cholesterol absorption [32,33], and this fraction also accounts for approximately 30% of total HDL content in the circulation [34] and protects the liver against inflammation by inactivating bacterial lipopolysaccharides from the intestines [35]. Moreover, HDL of intestinal origin has been demonstrated to be an important contributor to RCT, as determined in mice by intraperitoneally infusing macrophages loaded with labeled cholesterol and determining the amount of its excretion in feces [28,36]. However, the elegant demonstration of the cholesterol transit from the tissues to the intestinal lumen, the contribution of in vivo RCT, as well as the contribution of TACE to prevent coronary events remains to be demonstrated.

### 2.1. Pharmacological Elevation of HDL-C Does Not Support RCT as a Major Anti-Atherogenic Pathway

The paradigm of RCT seemed to conciliate the inverse relationship between levels of HDL cholesterol and CAD risk; consequently, increasing HDL-C has been an attractive target to prevent and treat the clinical manifestation of the disease. Under this premise, several drugs designed to increase the HDL-C plasma levels were proven, such as niacin, fibrates, and CETP inhibitors. Despite the significant increase in HDL-C plasma levels and the increased cholesterol efflux in vitro induced by such drugs [37,38,39,40], the expected reduction in coronary events was not observed in most of the studies. Table 1 briefly describes some of the major clinical trials on this topic and their main final interpretation.

### 2.2. Low Levels of HDL with Moderate or No Increased Risk of CAD

Patients with mutations in apo AI (i.e., apo AI_Milano_ and apo AI_Paris_), ABC-A1 (Tangier disease) and LCAT deficiency [50,51,52,53] have very low plasma concentrations of HDL cholesterol, but their CAD risk is often similar or slightly elevated compared to normal subjects [50,51,52,53]. Finally, normal plasma concentrations of HDL-C are observed in about half of patients with clinical manifestations of CAD, as demonstrated in the Framingham study [54].

All these studies show that the paradigm of RCT is not helpful in formulating adequate predictions concerning the risk of CAD manifestations. The putative beneficial role of HDL against atherosclerosis should be the consequence of a more fundamental function of these lipoproteins; in other words, HDL has not been conserved along the evolution to protect the arteries against atherosclerosis. Consequently, it is necessary to reconsider the biological role of HDL, from their evolutive origins to their participation in pathological processes.

## 3. Lipoprotein Evolution across Species

The presence of lipoproteins has been described in animals ranging from invertebrates to mammals. In insects, the lipoproteins are called lipophorins; these particles transport lipids from either exogenous or endogenous origin [55], and they are the main component of the hemolymph, the analog of mammals’ blood [55,56]. Lipophorin is a non-covalent spherical assembly of lipids and proteins [55]. The diacylglycerols (DAG) and hydrocarbons make up the core of lipophorins, whilst monolayer phospholipids at the surface play a structural role [55]. The protein fraction is integrated with apolipophorin I and apolipophorin II, which are non-exchangeable apolipoproteins [55,56,57]. Both are products of the post-translational cleavage of a precursor protein (apoLp-II/I) which belongs to the large lipid transfer protein superfamily [57]. A third protein forms part of this assembly; this protein is apolipophorin III (apoLpIII), which is an exchangeable protein. ApoLpIII is a protein that exists in both lipid-free and lipid-bound states [55,56,57]. The free form may act as a pathogen recognition receptor and stimulate the action of defense peptides [58]. On the other hand, the lipid-bound apoLpIII stabilizes lipophorins rich in DAG and is further released once they are captured for hydrolysis [59].

Four types of high-density lipophorins (HDLp) have been described in the moth *Manduca sexta* along the larval to pupal transition. The remodeling of HDLp is precisely timed, and it is associated possibly with metabolic requirements of particular moments in life stages [60]. In this context, slight similarities may be postulated between lipophorins and lipoproteins in humans; there are differences between fetal and adult HDL in humans. As detailed below, fetal HDL are rich in triglycerides and apo E. They are the main vehicle of cholesterol from de novo synthesis in the fetus [61], whilst HDL in adults is rich in phospholipids and apo AI [1].

In adult insects, lipophorins function as reusable lipid shuttles. HDLp are synthesized in the fat body, which has similar functions to the mammalian liver and adipose tissue [55,56,57,59]. HDLp receive DAG from the fat body, increasing their size and decreasing their density. In addition, apoLpIII is further incorporated into these lipophorins, which become low-density lipophorin (LDLp) because their buoyant density is similar to that of mammalian LDL [55]. Once the DAG has been delivered to flight muscles as an energy source, HDLp is regenerated to begin a new cycle of uptake and transport of lipids [55,59]. Again, the lipophorin metabolism maintains important similarities with HDL intravascular remodeling, thus suggesting that HDL emerged early in evolution as a bidirectional shuttle of lipids.

The presence of apo AI, the main apolipoprotein of adult HDL, has been described in a few evolved cartilaginous fish such as *Callorhinchus millii*, amphibians, i.e., *Xenopus tropicalis*, birds (*Gallus gallus*), and mammals such as *Canis lupus familiaris*, *Macaca mulatta*, and *Homo sapiens* [9]. The conservation of this protein among several species supports the hypothesis that apo AI plays a fundamental physiological role in organisms; other than to prevent atherosclerosis, the anti-atherogenic properties of HDL are secondary to their main physiological purpose. The ability of these particles to transport lipids may have had their origin in the cell membrane of prokaryotes [62]. Along with the evolution of multicellular organisms, lipids transported in aqueous environments between cells become indispensable to membrane structure and tissue repair; then, lipoproteins acquire such function. Therefore, it is likely that the fundamental function of HDL may be to act as lipid vectors between tissues, a role that becomes crucial during tissue repair, as discussed below.

## 4. HDL as Lipid Vectors

The main site of cholesterol synthesis is the liver, which produces about 50% of the total cholesterol in the body [63]. Then, cholesterol should reach the peripheral tissues packed in a lipoprotein, i.e., very-low-density lipoproteins (VLDL). The intravascular lipolysis of triglycerides contained in VLDL leads to LDL formation, which are the lipoproteins with the largest content of cholesterol in humans. Consequently, they have been considered the major cholesterol vehicle to the tissues [64]. However, there are important issues that do not support this hypothesis: (1) the main function of VLDL is the transport of triglycerides from the liver to the tissues, and these particles become enriched with cholesterol intravascularly; (2) the majority of the cholesterol from VLDL/LDL is returned to the liver; and (3) some tissues lack detectable uptake of cholesterol mediated by LDL-receptors [65]. This suggests when a cell’s cholesterol needs to increase, i.e., tissue repair, replication or growth, the quantity of cholesterol that it could receive from LDL would not be enough. Instead, the bi-directional HDL transporter SR-BI is widely expressed in most organism cells [66]. Moreover, besides VLDL, HDL is the other class of lipoproteins synthesized by the liver, suggesting that these lipoproteins may carry the hepatic cholesterol to extrahepatic tissues.

**Figure 2:**
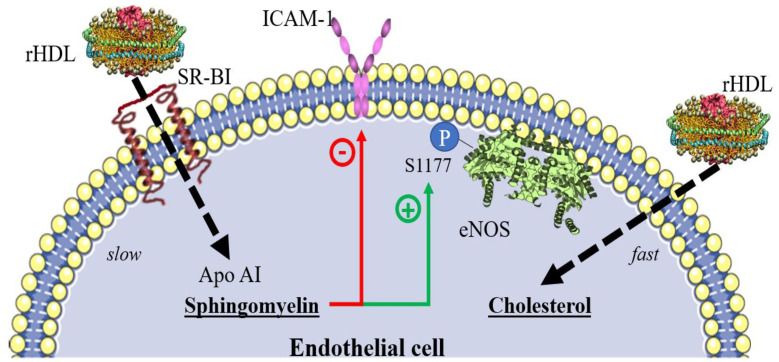
Lipids delivered to endothelial cells by HDL, according to Muñoz et al. [67]. Reconstituted HDL (rHDL) delivers cholesterol and sphingomyelin to HMEC-1 cells in culture. Cholesterol becomes integrated into the cells within the first 10 min in an SR-BI-independent manner. In contrast, SR-BI-dependent sphingomyelin and apo AI kinetics are slower, reaching the maximal delivery in about 30 min. The content of sphingomyelin in rHDL, but not of cholesterol, determines the TNF-induced expression of ICAM-1 and eNOS phosphorylation in the Ser 1177. VCAM-1 expression was not affected by any component of rHDL.

Results from our laboratory and other research groups support the hypothesis that HDL delivers lipids, probably from the hepatic origin, to cells [67]. We demonstrated that HDL delivers cholesterol and sphingomyelin to endothelial cells in culture [67]. Importantly, the kinetics of internalization of the former is faster than that of the latter; most of the cholesterol from HDL is integrated into the cells within the first 30 min of incubation in an SR-BI-independent manner. This cholesterol pathway may implicate other HDL receptors, such as the F(1)-ATPase/P2Y(13) complex [22,23]. In contrast, sphingomyelin is delivered after 30 min of incubation following the same internalization kinetics as apo AI [67]. These data suggest that cholesterol dissociates from HDL to be delivered to the cell, and the remaining particle is further internalized (Figure 2). The endothelial cells internalize cholesterol from HDL despite very high concentrations of LDL cholesterol [67]. These results suggest that the extrahepatic cells can take up cholesterol from HDL rather than from LDL (which requires the presence of ApoB receptors). They are consistent with earlier studies demonstrating that HDL inhibited LDL uptake by bovine endothelial cells [64,68,69].

Besides cholesterol, HDL delivers sphingomyelin to endothelial cells in culture, which mediates eNOS activation via phosphorylation and ICAM-1 expression [67]. These results suggest that some of the beneficial effects of HDL on vascular functions depend upon sphingomyelin. Taking into account the high complexity of HDL, which includes over 200 species of lipids and about 85 different proteins [70,71,72,73], the universe of possible effects of these lipoproteins on cell function after the internalization and delivery of their content is extremely high. In this context, the wide effects that have been attributed to HDL in health as well as in disease are more comprehensible [73,74].

Focusing only on lipid delivery, the contribution of HDL becomes of particular importance when cell membranes should be intensively synthesized or re-structured, i.e., during fetal development, tissue repair, intensive intracellular vesicle fusion with a plasmatic membrane, and cancer processes, as mentioned below.

### 4.1. The Role of HDL in Tissue Repair during Acute Phase Response and Inflammatory Processes

Besides the anti-inflammatory role of HDL from intestinal origin mentioned above [35], HDL-C plasma levels and composition may change drastically during inflammatory processes. A significant HDL-cholesterol level decrease is observed during sepsis [75], diffuse axonal injury [76], neural injury [77], and acute coronary syndrome [78], among others. In the same line of evidence, HDL protects against doxorubicin-induced cardiotoxicity in mice [79], whereas increased plasma levels of HDL induced by the CETP inhibitor des-fluoro-anacetrapib inhibits intimal hyperplasia in New Zealand White rabbits subjected to endothelial denudation of the abdominal aorta [80]; importantly, both effects were dependent of SR-BI.

The dramatic modifications of HDL structure during inflammation or tissue injury [81,82,83,84,85] strongly suggest a short-term rescue mechanism for cell survival when facing the insult. Besides the capacity of HDL to scavenge lipopolysaccharides produced during damage to the tissues driven by infectious processes [86,87], HDL seems to participate as carriers of lipids from dead cells after acute tissue injury [88,89]. Such lipids need to be recovered and reintegrated into the still viable cells and new cells for tissue repair. This role of HDL may be enhanced by amyloid A (AA) peptides [88]; during tissue injury, mediators of inflammation, i.e., IL-1β, and TNFα, induce the expression of serum AA, which becomes associated mainly with HDL [81]. The physiological role of amyloid A has not been completely understood but seems to lead HDL to the site of the injury [89,90]. It can also be speculated that AA fulfills the role of a transient apolipoprotein [91] intended to increase the capacity of HDL to deliver lipids to the cells via SR-BI [92]. SR-BI is one of the putative receptors for AA that induces HDL internalization [92]; congruently, HDL isolated from *Scarb1*-deficient mice (*SR-BI*^−/−^) are enriched in AA [93]. As described above, some of the HDL functions are mediated by their sphingomyelin content, and in turn, sphingomyelin is internalized to endothelial cells via SR-BI [67]. Then, AA may enhance the capacity of HDL to deliver functional and structural lipids to cells during the acute phase. In addition, the AA displaces some apolipoproteins [81,94], including apo AI [88], from HDL. The displaced apolipoproteins provide the opportunity of integrating supplementary HDL particles to manage the necessity of lipid transport and delivery during the acute phase. Importantly, the AA is a highly conserved protein along with evolution, similar to apo AI [9,88,90,95], suggesting a long-term adaptative interaction between both proteins.

The proposed role of HDL as a critical lipid vector for tissue repair after an injury is in agreement with several observations; as described above, patients with an acute coronary syndrome whose HDL-C plasma levels drop below 30 mg/dL had an odds ratio = 2.0 of intrahospital death [78]. Viable cells after the coronary event require rescue and repair, increasing the need for lipids for membrane restoration. The availability of such lipids in HDL helps promote the more efficient and faster recovery of damaged cells and, consequently, increases the possibility of survival. In the absence of enough lipid vectors, tissue repair would not be as fast as required to warrant the recovery of the organ function. The correct restoration of the endothelium in rabbits with increased HDL levels previously described [80] also supports this idea further. Whether the increase in HDL during the acute phase provides additional protection and helps repair tissues other than the cardiovascular system as suggested by previous reports [76,77,79,96] warrants future research.

### 4.2. HDL in Fetal Development

Embryogenesis and fetal development require large amounts of cholesterol and other lipids for normal development. The cholesterol of de novo synthesis in fetal cells is the main source of this lipid in the fetus [61]. The second source of fetal cholesterol is the mother; this exogenous cholesterol is transferred from the mother’s HDL to the syncytiotrophoblast of the placenta. Cholesterol is acquired from the maternal plasma HDL through the apical side of the syncytiotrophoblast layer, which expresses SR-BI [97]. This observation is consistent with the early described role of SR-BI in the internalization of cholesterol from HDL [21]. Then, the acquired cholesterol reaches the villous stroma and is transported by the endothelium of the fetal circulation. Accordingly, the fetuses of mice dams not expressing apo AI (Apoa1^−/−^) were 25% smaller than controls and had less cholesterol mass by fetus [98]. Importantly, the endogenous production of cholesterol by the fetus from Apoa1^-/-^ dams was comparable to that of controls, emphasizing the contribution of maternal HDL as cholesterol vectors to fetal development [98]. In the same context, Santander et al. [99] demonstrated that embryos lacking SR-BI exhibit a high prevalence of neural malformations and contain less cholesterol than normal littermates. Importantly, female mice deficient in SR-BI are infertile, probably due to abnormalities in the viability and developmental potential of their oocytes [100]. In addition, SR-BI-deficient pups exhibited intrauterine growth restrictions. The authors concluded that the SR-BI is involved in the maternal-fetal transport of cholesterol and/or other lipids with a role during neural tube closure and fetal growth [99].

Once the lipids from the mother or synthesized by the fetus reach the fetal circulation, they are transported and delivered mainly by HDL; during these stages of intense cell proliferation, more than 50% of the cholesterol and other lipids are contained in HDL [61,101,102]. Fetal HDL are larger than in adults, and they are particularly rich in apo E (for a review, see reference [61]). As expected, the intravascular metabolism of HDL in fetal circulation differs from that of adults; previous studies demonstrated that the activity of CETP is significantly lower in umbilical cord than in the mothers [99,100,101,102]. Taken together, the low CETP activities, the large HDL observed in fetal circulation, and the high impact of SR-BI receptor on embryo development [99], it is plausible to conclude that HDL functions as vectors of lipids for tissues during intrauterine development.

### 4.3. HDL in Cancer

Malignant cell survival requires large amounts of nutrients and lipids for membrane structure; consequently, cholesterol supply is needed for tumor development. The contribution of HDL to the growth of malignant cells is controversial and seems to depend on the type and localization of the tumor. Particularly, HDL can stimulate the growth of both estrogen-dependent and independent breast cancer cells in vitro [103]. Additionally, HDL induced the proliferation of androgen-independent prostate cancer cells [104]. These findings are consistent with an increased SR-BI expression in Leydig cell tumors, nasopharyngeal carcinoma, prostate cancers, and some breast cell lines such as HBL-100 and MCF-7 [105,106,107]. The role of SR-BI has been described mainly in breast and prostate cancers; for these tumors, the internalization of cholesterol from HDL via SR-BI enhances the tumor progression and aggressiveness [103,108]. Accordingly, with the preference for cholesterol from HDL in HMEC-1 cells [67], metastatic prostate tumors overexpress SR-BI receptors but not LDL receptors [108]. Consistently, down-regulation of SR-BI in prostate cell lines resulted in decreased cellular viability [109] and inhibition of motility of nasopharyngeal cancer cell lines [110]. These observations support the idea that one of the main physiological roles of HDL is to be carriers of lipids for cells in development. Since the mechanism of lipid delivery to the cells by HDL involves the internalization of the lipoprotein particle [16,17,67], it is reasonable to postulate cancer treatments with reconstituted HDL, including antitumoral molecules in their structure [105,111].

## 5. HDL Contribution to Insulin Secretion

The efflux of lipids is also a proven property of HDL that may play an important role in insulin secretion besides the importance of lipid influx promoted by HDL. Low plasma levels of HDL in type 2 diabetes mellitus have been considered a consequence more than a contributor to pancreatic β-cell dysfunction; increased triglyceride transfer from VLDL in coordination with hepatic lipase activity [2,112] and a high clearance rate of methylglyoxal-modified apo AI [113] have been accepted as some of the main causes of hypoalphalipoproteinemia in this physiopathological condition. However, there is increasing evidence for an important role of HDL in glucose homeostasis and insulin secretion by pancreatic β-cells [114,115,116]. Accordingly, a pharmacological increase of HDL with CETP inhibitors was associated with a significant rise in insulin plasma concentration [115] and with a significant risk reduction of new onset of diabetes in patients treated with dalcetrapib [117]. Since HDL has been demonstrated to promote cholesterol efflux from β-cells in culture [115,116,118], it has been argued that HDL prevents lipotoxicity induced by oxidized LDL and accumulation of cholesterol in β-cells [116,118]. However, there is no plausive evidence that demonstrates a cholesterol accumulation reaching toxic levels in β-cells in vivo.

In this context, it has been shown that about 9000 [119] insulin granules are contained in each β-cell, which is equivalent to more than 30 times the cell surface area. Every time plasma glucose concentrations increase, a large number of granules fuse with the cytoplasmatic membrane for insulin exocytosis. As a result, there is a constant cell surface expansion that should be compensated by continuous cytoplasmic membrane endocytosis [120,121]. In fact, when such endocytosis is impaired, the β-cell dysfunction is unable to secrete insulin in response to increased glucose concentrations, leading to glucose intolerance, as demonstrated in mice [121]. Thus, HDL may contribute to regulating and finely adjusting β-cell plasma membrane lipid composition [116] and insulin secretion by delivering sphingolipids [67,122,123].

It has been demonstrated that sphingomyelin-derived lipids, particularly sphingosine and sphingosine-1-phosphate, modulate the docking, Ca^2+^ sensitivity, and membrane fusion during exocytosis of granule contents [122,123]. As stated before, sphingomyelin may be delivered to cells by HDL [67], thus raising the possibility of contributing these lipoproteins to the β-cell function by maintaining its sphingomyelin supply. This explanation is consistent with recent reports that demonstrated an enhanced insulin secretion when MIN-6 β-cells were incubated with HDL [116]. The same study [116] demonstrated an increased cholesterol efflux promoted by HDL, as observed in several previous works with different types of cultured cells. It is important to emphasize that HDL promotes not only cholesterol but also phospholipid efflux from cultured cells [124,125]; in other words, HDL recovers membrane fragments from cells. Therefore, it is likely that HDL removes excess membrane lipids, i.e., from granule-mediated secretory cells, even if studies have been biased exclusively toward cholesterol efflux. Therefore, in addition to membrane endocytosis, HDL may contribute to compensate for the excess membrane lipids derived from vesicle fusion (exocytosis). Consistently with this idea, patients with Tangier disease are characterized by an impaired HDL-mediated lipid efflux [126,127] and concomitant glucose intolerance and decreased insulin secretion [128]. In the same vein, the ABC-A1 polymorphism rs9282541 that results in a substitution of arginine 230 for cysteine is associated with the increased incidence of type 2 diabetes mediated by HDL cholesterol plasma levels [129]. Finally, a recent meta-analysis demonstrated that CETP inhibitors decrease the risk of new-onset of diabetes by 16%, concomitantly with significant increases in HDL-C [130].

## 6. Conclusions

HDL has been conserved along with evolution, indicating a fundamental role for living organisms. Such a role should be congruent with their atheroprotective properties as well as their beneficial association with insulin secretion. The existing evidence demonstrates that one of the main functions of HDL is to act as bidirectional lipid carriers, delivering cholesterol and sphingomyelin, for example, to endothelial cells. The capacity of HDL to deliver lipids to the cells seems to be relevant after a tissue insult contributing to wound healing. Whether this mechanism is related to the protective role of HDL vis-à-vis the development of vascular damage during the first stages of atherosclerotic plaque development remains to be elucidated. In addition, the property of HDL to promote membrane lipid efflux could be particularly relevant to maintaining the functionality of vesicle-mediated secretory cells such as β-cells.

## Figures and Tables

**Table 1:** Examples of the main studies focusing on pharmacological HDL-C increase as a target to prevent coronary events.

Clinical Trial	Description	Results	Reference
Niacin			
AIM-HIGH	A total of 3414 patients with established atherosclerotic cardiovascular disease were randomly assigned to treatment with niacin (1.5–2 g/day) or placebo	LDL-C and triglycerides decreased by 16 and 26%, respectively. In contrast, HDL-C increased 20% in the niacin group. There was no benefit in reducing cardiovascular events, and the study was stopped for a mean follow-up of 3 years	[41]
HPS2-THRIVE	A total of 5673 patients with occlusive arterial disease were randomized into a niacin (2 g/day)/laropiprant (40 mg/day) and placebo group	An increase in 6 mg/dL in the HDL-C was observed in the niacin group. The niacin/laropiprant combination did not decrease the incidence of main vascular events but increased the risk of serious adverse events. The study was stopped after 3.9 years	[42]
Fibrates			
HHS	A total of 4081 asymptomatic men with primary dyslipidemia and without cardiovascular disease were divided to receive 1.2 g/day of gemfibrozil or a placebo	The reduction of total cholesterol (10%), LDL-C (11%), triglycerides (35%) and non-HDL cholesterol (11%), as well as an increase in HDL-C (10%) in the gemfibrozil group, were the main lipid findings. Fatal and non-fatal MI or cardiac death showed a reduction of 34%	[43]
BIP	A total of 3090 patients with CAD, HDL-C <45 mg/dL and moderately elevated total cholesterol received 400 mg/day of bezafibrate or placebo	HDL-C increased 18%, and triglycerides decreased 21% in the bezafibrate group. The CAD mortality and non-fatal myocardial infarction were similar in both groups. Treated patients with baseline triglycerides ≥200 mg/dL presented a reduced cumulative probability of a primary endpoint (fatal or non-fatal myocardial infarction or sudden death) by 39%. High HDL-C levels did not reduce the cumulative probability of a primary endpoint	[44]
FIELD	A total of 9795 patients with T2D were treated with 200 mg/day of fenofibrate or placebo	Triglycerides, total cholesterol, and LDL-cholesterol were reduced by 21.9%, 6.9% and 5.8%, respectively, whilst HDL-C increased by 1.2%. Fenofibrate did not reduce the risk of the primary outcome of coronary events (coronary heart disease death or non-fatal myocardial infarction)	[45]
Inhibitors of CETP			
ILLUMINATE	A total of 15,067 patients at high cardiovascular risk underwent randomization and received torcetrapib (60 mg/day) or placebo	Torcetrapib increased HDL-C by 72.1% and decreased LDL-C levels by 24.9% but was associated with an increased death risk of both cardiovascular and non-cardiovascular causes. The study was stopped after a follow-up of 18 months	[46]
dal-OUTCOMES	A total of 15,871 patients with a recent ACS received dalcetrapib (600 mg/day) or placebo	Dalcetrapib increased HDL-C and apo A-I concentrations by 35% and9%, respectively. Minimal effects on LDL-C and apolipoprotein B were observed. Treatment did not decrease the risk of recurrent cardiovascular events, and the study was interrupted after a follow-up of 31 months	[47]
ACCELERATE	A total of 12,092 patients with ACS, peripheral vascular arterial disease, atherosclerotic cerebrovascular disease, or T2D with CAD were treated with evacetrapib (130 mg/day) or a placebo	HDL-C levels increased by 133.2%, and LDL-C levels reduced by 31.1% in the evacetrapib group. Evacetrapib was not associated with reducing the death risk from cardiovascular disease. Due to the lack of efficacity, the trial was terminated after a follow-up of 28 months	[48]
REVEAL	A total of 30,449 patients at high risk for cardiovascular events were randomized to receive anacetrapib (100 mg/day) or a placebo	Lower levels of LDL-C by 41% and non-HDL cholesterol by 18% and higher levels of HDL-C by 104% were observed in the anacetrapib group compared to placebo. There was a lower rate of major coronary events, but there was no difference in the risk of coronary death. Increases in HDL-C did not have a large effect on coronary events	[49]

AIM-HIGH: Atherothrombosis Intervention in Metabolic Syndrome with Low HDL/High Triglycerides and Impact on Global Health Outcomes; HPS2-THRIVE; Heart Protection Study 2-Treatment of HDL to Reduce the Incidence of Vascular Events; HHS: Helsinki Heart Study; BIP: Bezafibrate Infarction Prevention; FIELD: Fenofibrate Intervention and Event Lowering in Diabetes; ILLUMINATE: Investigation of Lipid Level Management to Understand its Impact in Atherosclerotic Events; ACCELERATE: Assessment of Clinical Effects of Cholesteryl Ester Transfer Protein Inhibition with Evacetrapib in Patients at High Risk for Vascular Outcomes; REVEAL: Randomized Evaluation of the Effects of Anacetrapib through Lipid Modification; CAD: coronary artery disease; HDL-C: high-density lipoprotein-cholesterol; LDL-C: low-density lipoprotein-cholesterol; MI: myocardial infarction; T2D: type 2 diabetes mellitus; ACS: acute coronary syndrome; apo: apolipoprotein.
